# Editorial: Advanced fNIRS applications in neuroscience and neurological disorders

**DOI:** 10.3389/fneur.2025.1686834

**Published:** 2025-09-12

**Authors:** Huiting Qiao, Dan Zhang, Wentian Dong, Daifa Wang

**Affiliations:** ^1^Key Laboratory of Innovation and Transformation of Advanced Medical Devices, Ministry of Industry and Information Technology, National Medical Innovation Platform for Industry-Education Integration in Advanced Medical Devices (Interdiscipline of Medicine and Engineering), School of Biological Science and Medical Engineering, Beihang University, Beijing, China; ^2^Department of Psychological and Cognitive Sciences, Tsinghua University, Beijing, China; ^3^Peking University Sixth Hospital, Peking University Institute of Mental Health, NHC Key Laboratory of Mental Health (Peking University), National Clinical Research Center for Mental Disorders (Peking University Sixth Hospital), Beijing, China

**Keywords:** fNIRS application, neurodevelopment, cognitive function, disorders of consciousness, rehabilitation, psychiatry

Functional near-infrared spectroscopy (fNIRS) has rapidly emerged as a powerful neuroimaging tool that offers unique advantages for the investigation of brain function and neurological disorders. Using near-infrared light, fNIRS monitors changes in cortical blood oxygenation that reflect cortical neural activity and enable researchers and clinicians to study brain function in real time and in a non-invasive manner. Unlike many conventional imaging techniques, fNIRS is available in portable and wearable forms, making it suitable for use in real-world settings. The technology is highly resistant to motion artifacts and electromagnetic interference, imposing minimal constraints on the testing environment. As a result, fNIRS can be employed at the bedside or in clinical rehabilitation environments, even with patients who have difficulty remaining still or who have implants, such as pacemakers, that would typically prevent neuroimaging. Owing to these features, fNIRS opens up unparalleled opportunities to study the human brain in action. It deepens our understanding of neural mechanisms in both health and disease, and holds promise for earlier diagnoses and more personalized, precision medicine approaches in neurology.

This Research Topic aims to showcase recent advancements in the application of fNIRS in the fields of neuroscience and neurological disorders. A total number of 22 articles have been included: they highlight the utility of fNIRS in probing brain function and cognitive neural mechanisms, along with its clinical relevance in the diagnosis, assessment, mechanistic understanding and evaluation of rehabilitation outcomes in neurological conditions. The findings presented in this Research Topic not only affirm fNIRS as a powerful neuroimaging tool but also underscore its potential to advance diagnostic precision, therapeutic monitoring, and personalized interventions in the management of neurological disorders.

The collected articles underscore the remarkable versatility of fNIRS, which has been applied to a broad range of neurological and psychiatric conditions. On the neurodevelopmental front, a comprehensive review in this Research Topic surveyed fNIRS studies on numerous childhood disorders, including cerebral palsy, autism spectrum disorder, attention-deficit/hyperactivity disorder (ADHD), and neonatal brain injuries (Wang, Zou, Huang, Zhang, et al.). In another original study, Bian et al. used fNIRS to monitor prefrontal cortex activity during verbal fluency tasks in children with ADHD. In a separate original study in this Research Topic, Wang, Zou, Huang, Wu, et al. employed fNIRS to investigate its therapeutic effects, reporting that repetitive transcranial magnetic stimulation (rTMS) combined with standard care led to greater improvements in ADHD symptoms and prefrontal connectivity than standard care alone. The unique advantages of fNIRS—such as safety, non-invasiveness, wearability, compatibility with other modalities, and tolerance for movement—make it especially suitable for pediatric populations, enabling safe and reliable monitoring of neural changes even in young or inattentive children undergoing neuromodulation (Gao et al.).

In another domain, cognitive function and impairment stand to benefit from fNIRS innovations. Liu et al. explored healthy cognitive processing using fNIRS, showing that binocular color fusion—a demanding visual task—elicited significantly stronger prefrontal connectivity and higher activation than binocular rivalry. The study indicates that fNIRS can accurately track advanced perceptual–cognitive processes. Ruan et al. demonstrated that combining fNIRS-derived cortical oxygenation features with plasma biomarkers markedly improved the ability to distinguish Alzheimer's disease from Lewy body dementia in older adults. [Choi et al.] used fNIRS as a cognitive assessment tool and found that a 6-month herbal medicine and acupuncture program for patients with mild cognitive impairment led to cognitive improvement accompanied by increased prefrontal activation. Given its strong motion-tolerant capability for monitoring brain activity during complex perceptual tasks, fNIRS was also applied to assess cortical activation in stroke survivors performing combined balance and cognitive tasks (He et al.). These results highlight fNIRS's ability to evaluate how cognitive load influences motor networks in rehabilitation scenarios, while allowing patients to perform upright activities that would be infeasible inside traditional neuroimaging scanners. Yang and Wang further noted that fNIRS's high temporal resolution and portability complement fMRI's spatial precision. These combined approaches enable robust brain mapping and extend neuroimaging to populations or settings that are impractical for fMRI alone, which is particularly valuable for researching cognitive mechanisms.

Several contributions focused on using fNIRS in cases of severe brain injury and coma, areas in which traditional assessments are ineffective. Disorders of consciousness—such as coma, vegetative state, and minimally conscious state—present a diagnostic challenge because patients cannot communicate. Liang et al. used resting-state fNIRS to show that patients who later regained higher levels of consciousness exhibited stronger frontal–occipital connectivity than those who remained in a vegetative state, demonstrating the value of the technique for noninvasively probing residual brain networks and predicting outcomes. Zhang T. et al. applied fNIRS to assess pain processing in patients with disorders of consciousness and found minimal overt cortical activation but markedly enhanced connectivity among somatosensory, motor, and prefrontal areas. This illustrates the technique's sensitivity to coordinated network responses even in low-cooperative patients. [Wang N. et al.] reviewed fNIRS applications in disorders of consciousness, highlighting its portability, real-time monitoring capability, and compatibility with interventions such as brain–computer interfaces and neuromodulation. Zhao et al. described a neuromodulation trial protocol that employs fNIRS alongside EEG and behavioral assessments to track therapy-induced brain changes, underscoring its role as a safe, repeatable bedside tool for patients who are unsuitable for conventional neuroimaging.

In the field of rehabilitation, fNIRS enables objective, motion-tolerant monitoring of brain activity in real-world therapeutic contexts. Zhang, Wang, et al. demonstrated that, in contrast to clinical scales, fNIRS can quantitatively evaluate rehabilitation efficacy, with increased cortical oxygenation and connectivity providing neurophysiological support for the effectiveness of post-stroke spasticity interventions. Xiao et al. showed that, despite normal behavioral balance metrics, fNIRS can detect subtle central changes and compensatory strategies in musculoskeletal pain disorders, offering a more sensitive indicator of postural control deficits than standard measures. Xu et al. reported that functional electrical stimulation during walking in hemiparetic stroke patients reduced contralesional premotor cortex activation, illustrating how wearable fNIRS can capture gait-related neural changes in over-ground settings. Expanding its application to orthopedic rehabilitation, Cao et al. used wireless fNIRS to reveal reduced frontal–parietal activation during stair climbing in patients 3 months after anterior cruciate ligament reconstruction, suggesting neuroplastic changes following knee injury.

In the field of psychiatry, fNIRS offers a non-invasive, portable, and task-compatible means of detecting disorder-specific neural alterations in populations that are often difficult to assess with conventional neuroimaging techniques. Zhang, Tian, et al. showed that patients with schizophrenia who display auditory hallucinations exhibit altered frontotemporal activation patterns during verbal fluency tasks, demonstrating fNIRS's ability to capture symptom-specific brain dysfunction. Ding et al. reported that patients with depression, anxiety, or insomnia exhibit distinct reductions in task-based prefrontal connectivity, supporting the use of fNIRS as a tool for differentiating executive deficits across heterogeneous psychiatric disorders. Chen D. et al. developed a free-association semantic task for perinatal depression, revealing that prefrontal oxyhemoglobin changes correlate with symptom severity. This highlights the applicability of fNIRS in low-cooperative populations. In postpartum women, Chen X. et al. further linked insomnia-related disruptions in prefrontal–temporal connectivity and network efficiency to mood symptoms, illustrating how real-time, portable fNIRS monitoring can reveal subclinical brain changes outside of traditional laboratory environments.

Beyond clinical and laboratory settings, Si et al. demonstrated the feasibility of using a wearable fNIRS oximeter for continuous monitoring of cerebral oxygen saturation in participants during high-altitude expeditions. This type of monitoring is critical for preventing brain injuries associated with both acute and chronic high-altitude exposure. The findings highlight the robustness of fNIRS under extreme, mobile conditions and its potential for managing brain oxygenation during environmental or exercise-related challenges, thereby extending neuromonitoring far beyond the confines of the clinic.

Taken together, the contributions to *Advanced fNIRS Applications in Neuroscience and Neurological Disorders* demonstrate that fNIRS has become a mainstream neuroimaging modality. No longer a niche technique, it is now shedding new light on brain function across ages and disorders—from illuminating the underpinnings of childhood ADHD and autism, to tracking recovery in stroke and disorders of consciousness, to augmenting our ability to diagnose subtle cognitive impairment. The key message of this editorial collection is that fNIRS's non-invasiveness and portability are more than mere conveniences—they are gateways to groundbreaking research avenues and transformative clinical applications. In the years ahead, fNIRS is poised to play an increasingly vital role in both research and clinical practice, driving progress in brain science and improving outcomes for individuals with neurological and psychiatric conditions.

